# Determining prognostic indicator for anticoagulant therapy in sepsis-induced disseminated intravascular coagulation

**DOI:** 10.1186/s40560-024-00739-x

**Published:** 2024-06-24

**Authors:** Toshiaki Iba, Kazuma Yamakawa, Yuki Shiko, Ryo Hisamune, Tomoki Tanigawa, Julie Helms, Jerrold H. Levy

**Affiliations:** 1 Department of Emergency and Disaster Medicine, Juntendo University Graduate School of Medicine, 2-1-1 Hongo Bunkyo-Ku, Tokyo, 113-8421 Japan; 2 Department of Emergency and Critical Care Medicine, Osaka Medical and Pharmaceutical University, Osaka, Japan; 3 Department of Biostatistics, Graduate School of Medicine, Research Administration Center, Saitama Medical University, Saitama, Japan; 4 Medical Affairs Section, Research and Development Division, Japan Blood Products Organization, Tokyo, Japan; 5 Strasbourg University (UNISTRA); Strasbourg University Hospital, Medical Intensive Care Unit, NHC; INSERM (French National Institute of Health and Medical Research), UMR 1260, Regenerative Nanomedicine (RNM), FMTS, Strasbourg, France; 6 Department of Anesthesiology, Critical Care, and Surgery, Duke University School of Medicine, Durham, NC USA

**Keywords:** Disseminated intravascular coagulation, Sepsis, Antithrombin, Sequential Organ Failure Assessment score, Endpoint

## Abstract

**Background:**

There is no reliable indicator that can assess the treatment effect of anticoagulant therapy for sepsis-associated disseminated intravascular coagulation (DIC) in the short term. The aim of this study is to develop and validate a prognostic index identifying 28-day mortality in septic DIC patients treated with antithrombin concentrate after a 3-day treatment.

**Methods:**

The cohort for derivation was established utilizing the dataset from post-marketing surveys, while the cohort for validation was acquired from Japan’s nationwide sepsis registry data. Through univariate and multivariate analyses, variables that were independently associated with 28-day mortality were identified within the derivation cohort. Risk variables were then assigned a weighted score based on the risk prediction function, leading to the development of a composite index. Subsequently, the area under the receiver operating characteristic curve (AUROC). 28-day survival was compared by Kaplan–Meier analysis.

**Results:**

In the derivation cohort, 252 (16.9%) of the 1492 patients deceased within 28 days. Multivariable analysis identified DIC resolution (hazard ratio [HR]: 0.31, 95% confidence interval [CI]: 0.22–0.45, *P* < 0.0001) and rate of Sequential Organ Failure Assessment (SOFA) score change (HR: 0.42, 95% CI: 0.36–0.50, *P* < 0.0001) were identified as independent predictors of death. The composite prognostic index (CPI) was constructed as DIC resolution (yes: 1, no: 0) + rate of SOFA score change (Day 0 SOFA score–Day 3 SOFA score/Day 0 SOFA score). When the CPI is higher than 0.19, the patients are judged to survive. Concerning the derivation cohort, AUROC for survival was 0.76. As for the validation cohort, AUROC was 0.71.

**Conclusion:**

CPI can predict the 28-day survival of septic patients with DIC who have undergone antithrombin treatment. It is simple and easy to calculate and will be useful in practice.

**Supplementary Information:**

The online version contains supplementary material available at 10.1186/s40560-024-00739-x.

## Introduction

Disseminated intravascular coagulation (DIC) is a frequently acquired condition following sepsis, which is associated with high mortality rates. A prospective survey conducted in Japan reported that the prevalence of DIC was 50.9%. Patients with DIC exhibited a higher prevalence of Multiple Organ Dysfunction Syndrome (MODS) (32.0% vs. 13.1%) and a worse mortality rate (24.8% vs. 17.5%) compared to patients without DIC [1]. DIC arises from dysregulated coagulation and fibrinolytic systems, closely intertwined with systemic inflammation. The disseminated microthrombosis with systemic microvascular impairment culminates in multiple organ damage due to tissue malcirculation [2], of which thrombin is a pivotal mediator that facilitates coagulation and inflammation described as thromboinflammation [3].

Antithrombin is the most abundant physiological anticoagulant that inhibits multiple coagulation factors, including thrombin [4]. This forms the basis for the potential efficacy of antithrombin repletion in consumptive coagulopathies, including DIC. Although meta-analyses of the randomized controlled trials (RCTs) did not show significant differences in mortality, that of observational studies indicated a trend of decreasing mortality rates with antithrombin administration (odds ratio [OR]: 0.79, 95% confidence interval [CI]: 0.68–0.92, *P* = 0.002) [5]. Tagami et al. [6] performed a propensity score-matched analysis on a Japanese nationwide administrative database. They created a matched cohort of 2194 pairs from 9075 severe pneumonia patients and found 28-day mortality was lower in patients treated by antithrombin (40.6% vs. 44.2%, adjusted OR: 0.85, 95% CI: 0.75–0.97).

Although antithrombin is commonly used for the treatment of sepsis-induced DIC in Japan, there is no clinically available indicator to judge its efficacy in the short term. The aim of this study is to develop and validate a prognostic index identifying 28-day mortality in septic DIC patients treated with antithrombin concentrate after a 3-day treatment. We expect a composite indicator built on multiple factors, such as improvement in organ dysfunction and DIC score, to be helpful for that purpose [7]. The advantage of this evaluation method is that it allows us to design the endpoint to assess treatment effects within a relatively short time period [8].

## Materials and methods

### Study design and data

This observational study utilized two distinct cohorts. The derivation cohort was drawn from a post-marketing survey of the antithrombin concentrate (Neuart^®^; Japan Blood Products Organization, Tokyo), while the validation cohort was derived from the Japan Septic Disseminated Intravascular Coagulation (JSEPTIC-DIC) study (UMIN-CTR ID: UMIN000012543) [9]. The Japan Blood Products Organization conducted the post-marketing survey from April 2013 to April 2016 across 213 hospitals in Japan gathered data on 2588 DIC patients based on the Japanese Association for Acute Medicine (JAAM) DIC criteria [10]. The JSEPTIC-DIC study, a nationwide multicenter retrospective cohort study in 42 intensive care units (ICUs) from January 2011 to December 2013, collected data on 3195 patients with severe sepsis or septic shock.

### Participants

**Fig. 1:**
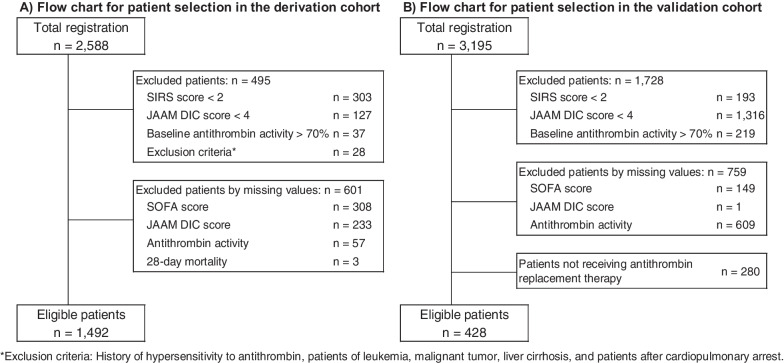
Flowchart for patient selection. **A** Flowchart for patient selection in the derivation cohort. From 2588 cases in post-marketing surveillance for antithrombin concentrate, 1,492 data sets that contain the necessary data on Days 0 and 3 were selected and served for derivation. **B** Flowchart for patient selection in the validation cohort. From the 3915 data sets in the JSEPTIC-DIC study, 428 that contain Days 0 and 2 were extracted and served for validation. JSEPTIC-DIC: the Japan Septic Disseminated Intravascular Coagulation, SIRS: systematic inflammatory response syndrome, JAAM DIC: Japanese Association for Acute Medicine disseminated intravascular disease, SOFA: Sequential Organ Failure Assessment

The study focused on the patients with sepsis-associated DIC who received antithrombin substitution. Inclusion criteria were infection-based DIC (JAAM DIC score of 4 or more) and antithrombin activity of 70% or less (Fig. 1). Participants with missing data on SOFA scores, JAAM DIC scores, antithrombin activity, or 28-day mortality were excluded. In the derivation cohort, patients with known allergies to antithrombin, leukemia, malignant tumors, liver cirrhosis, or post-cardiopulmonary arrest were also excluded. Antithrombin substitution dosage and duration were based on clinical judgment, typically adhering to the recommended 1500 IU/day dose for three days. Concomitant use of other anticoagulants was permitted. The primary outcome was the 28-day survival.

### Data collection

For the derivation cohort, data were collected at baseline before antithrombin substitution (Day 0) and on the day after a 3-day substation (Day 3). For the validation cohort, data were collected on Day 0, the first day in the ICU before the treatment, and Day 2.

To construct the prognostic index, we used baseline parameters: patient demographics, infection site, antithrombin activity, JAAM DIC score, SOFA score, antithrombin dosage, and usage of concomitant therapies. Variables were extracted from these items and selected based on their relevance in DIC risk stratification, as examined in a previous study [11]. In that study, Cox multivariate regression analysis, Kaplan–Meier curve analysis, and receiver operating characteristic (ROC) curve analysis were performed to evaluate the performance of variables used to assess mortality in the same cohort. Since detailed information, such as dosage and timing of administration, regarding other anticoagulants (e.g., heparin, protease inhibitors, and thrombomodulin) were lacking, they were not included in the present analysis. Additionally, the JAAM DIC score, SOFA score, and antithrombin activity on Day 3 were also utilized for derivation. Again, based on the previous studies [12–14], the post-treatment antithrombin activity, changes in JAAM DIC, and changes in SOFA score were served for analyses because they were identified as relevant to the outcome. Meanwhile, those data on Day 2 were used for validation.

### Statistical analysis

Continuous variables were expressed as mean ± standard deviation. Categorical data were presented as absolute numbers and percentages. Continuous variables were compared using Student’s t-test. Categorical variables were compared using Pearson’s Chi-square or Fisher’s exact tests. Univariate and multivariate Cox proportional-hazards regression analyses for 28-day mortality were performed and estimated the hazard ratio (HR) and 95% confidence interval (CI) for each variable.

We constructed a model to predict mortality at 28 days using a multivariate Cox proportional-hazards regression model. The evaluation of the model was conducted based on two concepts: calibration and discrimination. Calibration was assessed by creating a calibration plot to evaluate the conformity of predicted probabilities to actual probabilities, while discrimination was evaluated using the area under the receiver operating characteristic (AUROC) curve. Next, we calculated risk scores based on the regression coefficients of the multivariate model. We also determined the cutoff value for the risk score using Youden’s score and examined the discriminatory ability when the population was stratified based on this cutoff value. Finally, we conducted external validation of the developed model in a similar manner to assess its external validity. *P* value < 0.05 was considered statistically significant.

All statistical analyses were performed using the SAS statistical software package version 9.4 (SAS Institute, Cary, USA).

### Ethical approval

The study received ethical approval from the Institutional Review Board of Juntendo University, the Japan Blood Products Organization, and Osaka General Medical Center (#25-2050). Conducted in accordance with the principles of the Declaration of Helsinki, the post-marketing survey adhered to Good Post-Marketing Study Practice. As this research involved secondary data analysis of anonymized data, the requirement for informed consent was waived.

## Results

In the derivation cohort of 1492 patients, 1240 patients (83.1%) survived, while 252 patients (16.9%) died. The proportion of females was significantly higher among the survivors (*P* = 0.0049). Also, survivors were significantly younger (*P* < 0.0001), had higher antithrombin activity (*P* < 0.0001), and had a lower average SOFA score (8.9 vs. 11.8, *P* < 0.0001). However, the JAAM DIC score was not significantly different between survivors and non-survivors. Fewer survivors required renal replacement therapy or mechanical ventilation (*P* < 0.0001, respectively).

In the validation cohort of 428 patients, 345 (80.6%) survived, and 83 (19.4%) did not. Similar to the derivation cohort, survivors were significantly younger (*P* = 0.0331) and had a lower SOFA score (11.3 vs. 12.6, *P* = 0.0028), but there was no significant difference in the JAAM DIC score or antithrombin activity between survivors and non-survivors. Also, fewer survivors required mechanical ventilation (*P* = 0.0011) (Table 1).

**Table 1:** Baseline characteristics in the two cohorts

Variable	Derivation			Validation		
	Non-survivor *n* = 252 (%)	Survivor *n* = 1240 (%)	*P* value	Non-survivor *n* = 83 (%)	Survivor *n* = 345 (%)	*P* value
Sex			0.0049			0.9815
Female	83 (32.9)	528 (42.6)		35 (42.2)	145 (42.0)	
Male	169 (67.1)	712 (57.4)		48 (57.8)	200 (58.0)	
Age	73.4 (13.2)	69.8 (15.4)	< 0.0001	71.6 (14.2)	67.9 (14.1)	0.0331
Body weight	56.3 (15.1)	55.6 (13.2)	0.5587	55.6 (12.3)	57.2 (14.1)	0.3555
Antithrombin activity (%)	43.5 (12.9)	48.4 (12.2)	< 0.0001	46.0 (13.7)	47.3 (13.2)	0.4437
JAAM DIC score	5.6 (1.3)	5.6 (1.4)	0.7838	6.3 (1.4)	6.1 (1.4)	0.4895
SIRS	0.7 (0.4)	0.8 (0.4)	0.2293	0.8 (0.4)	0.8 (0.4)	0.7256
Platelet	2.1 (1.2)	2.0 (1.2)	0.0858	2.4 (1.0)	2.4 (1.0)	0.9432
PT-INR	0.9 (0.3)	0.8 (0.4)	0.0044	0.9 (0.3)	0.9 (0.3)	0.9033
FDP	1.8 (1.2)	2.0 (1.2)	0.0820	2.2 (1.1)	2.1 (1.1)	0.3805
SOFA score	11.8 (3.9)	8.9 (3.7)	< 0.0001	12.6 (3.5)	11.3 (3.5)	0.0028
Respiration	2.3 (1.2)	1.7 (1.2)	< 0.0001	2.3 (1.3)	2.1 (1.2)	0.1866
Coagulation	2.1 (1.1)	1.8 (1.1)	0.0029	1.9 (1.3)	1.9 (1.2)	0.6331
Liver	0.9 (1.1)	0.7 (0.9)	0.0018	0.8 (0.9)	0.9 (1.1)	0.2681
Cardiovascular	2.7 (1.5)	2.1 (1.7)	< 0.0001	3.1 (1.3)	2.9 (1.4)	0.2443
GCS	2.3 (1.5)	1.4 (1.4)	< 0.0001	2.1 (1.3)	1.4 (1.3)	< 0.0001
Renal	1.6 (1.3)	1.2 (1.3)	0.0001	2.4 (1.3)	2.1 (1.3)	0.0155
Antithrombin concentrate						
Daily dose (IU)	1594.7 (434.5)	1576.0 (457.7)	0.5498			
Duration (day)	3.5 (1.8)	3.2 (1.9)	0.0157			
Co-administration						
Heparin	45 (17.9)	188 (15.2)	0.2951	5 (6.0)	29 (8.4)	0.4713
Protease inhibitor	51 (20.2)	254 (20.5)	1.0000	21 (25.3)	86 (24.9)	0.9437
Thrombomodulin	154 (61.1)	711 (57.3)	0.2938	42 (50.6)	194 (56.2)	0.3545
Renal replacement therapy	122 (48.4)	332 (26.8)	< 0.0001	44 (53.0)	151 (43.8)	0.1290
PMX-DHP	35 (13.9)	191 (15.4)	0.6298	35 (42.2)	145 (42.0)	0.9815
Mechanical ventilation	185 (73.4)	623 (50.2)	< 0.0001	76 (91.6)	256 (74.2)	0.0011

Continuous variables are presented as means with standard deviation (SD), and categorical variables are presented as numbers with percentages

*JAAM* Japanese Association for Acute Medicine, *DIC* disseminated intravascular disease, *SIRS* systemic inflammatory response syndrome, *PT-INR* prothrombin time-international normalized ratio, *FDP* fibrin/fibrinogen degradation products, *SOFA* Sequential Organ Failure Assessment, *GCS* Glasgow Coma Scale, *IU* international unit, *PMX-DHP* direct hemoperfusion with Polymyxin B immobilized fiber

Multivariate Cox regression analysis following univariate regression demonstrated that sex, age, antithrombin activity on Day 3, changes in JAAM DIC score, and changes in SOFA score were significant factors that influenced survival (Table 2).

**Table 2:** Univariate and multivariate Cox regression analysis in the derivation cohort

Variable	Univariate analysis		Multivariate analysis	
	HR (95% CI)	*P* value	HR (95% CI)	*P* value
Male	1.46 (1.12, 1.90)	0.005	1.53 (1.15, 2.04)	0.004
Age	1.02 (1.01, 1.03)	0.001	1.02 (1.01, 1.03)	0.001
Antithrombin activity (Day3)	0.98 (0.98, 0.99)	< 0.0001	0.99 (0.99, 1.00)	0.020
Delta JAAM DIC score	0.74 (0.70, 0.79)	< 0.0001	0.83 (0.77, 0.89)	< 0.0001
Delta SIRS	0.52 (0.42, 0.63)	< 0.0001		
Delta PT-INR	0.45 (0.35, 0.57)	< 0.0001		
Delta FDP	0.89 (0.81, 0.98)	0.975		
Delta SOFA score	0.39 (0.33, 0.46)	< 0.0001	0.45 (0.38, 0.55)	< 0.0001
Delta respiration	0.75 (0.66, 0.84)	< 0.0001		
Delta coagulation	0.72 (0.64, 0.80)	< 0.0001		
Delta liver	0.61 (0.53, 0.70)	< 0.0001		
Delta cardiovascular	0.73 (0.67, 0.79)	< 0.0001		
Delta GCS	0.70 (0.63, 0.78)	< 0.0001		
Delta renal	0.75 (0.66, 0.86)	< 0.0001		

Delta = Day0–Day3

*JAAM* Japanese Association for Acute Medicine, *DIC* disseminated intravascular disease, *SIRS* systematic inflammatory response syndrome, *PT-INR* prothrombin time-international normalized ratio, *FDP* fibrin/fibrinogen degradation products, *SOFA* Sequential Organ Failure Assessment, *GCS* Glasgow Coma Scale, *HR* hazard ratio, *CI* confidence interval

For determining the prognostic index, since sex and age were unaffected by the treatment, they were not included in the index. In addition, although the change in JAAM DIC score and post-treatment antithrombin activity were found to be statistically significant in the multivariate analysis, they were not included in the prognostic index because their significance was smaller in the provisional calculation. With respect to the change in JAAM DIC score, when DIC resolution (DIC was considered resolved when the JAAM DIC scores decreased to less than four) was replaced with a change in JAAM DIC score, AUROC decreased from 0.76 to 0.74. In addition, as previously reported, the significance of the change in the JAAM DIC score was lower than that of a change in the SOFA score [13]. Since DIC resolution showed better performance and simplified the calculation, we adopted it for the CPI. Meanwhile, multivariate Cox regression identified DIC resolution (estimate: − 1.16, HR: 0.31, 95% CI: 0.22–0.45) and the rate of SOFA score change (estimate: − 0.86, HR: 0.42, 95% CI: 0.36–0.50) as independent predictors of mortality (Table 3). Based on an estimate of the Cox regression coefficient, both variables received an equal weight of 1. The rate of SOFA score change was calculated as (Day 0 SOFA score–Day 3 SOFA score) / Day 0 SOFA score. The composite prognostic index (CPI) = DIC resolution (yes: 1, no: 0) + rate of SOFA score change.

**Table 3:** Multivariate Cox regression for determining prognostic indicators in the derivation cohort

Variable	Estimate	SE	HR (95% CI)	*P* value
DIC resolution (Ref. = No)	− 1.16	0.19	0.31 (0.22, 0.45)	< 0.0001
Rate of SOFA score change*	− 0.86	0.09	0.42 (0.36, 0.50)	< 0.0001

*DIC* disseminated intravascular disease, *SOFA* Sequential Organ Failure Assessment, *SE* standard error, *HR* hazard ratio, *CI* confidence interval

*(Day 0 SOFA score–Day 3 SOFA score) / Day 0 SOFA score

**Fig. 2:**
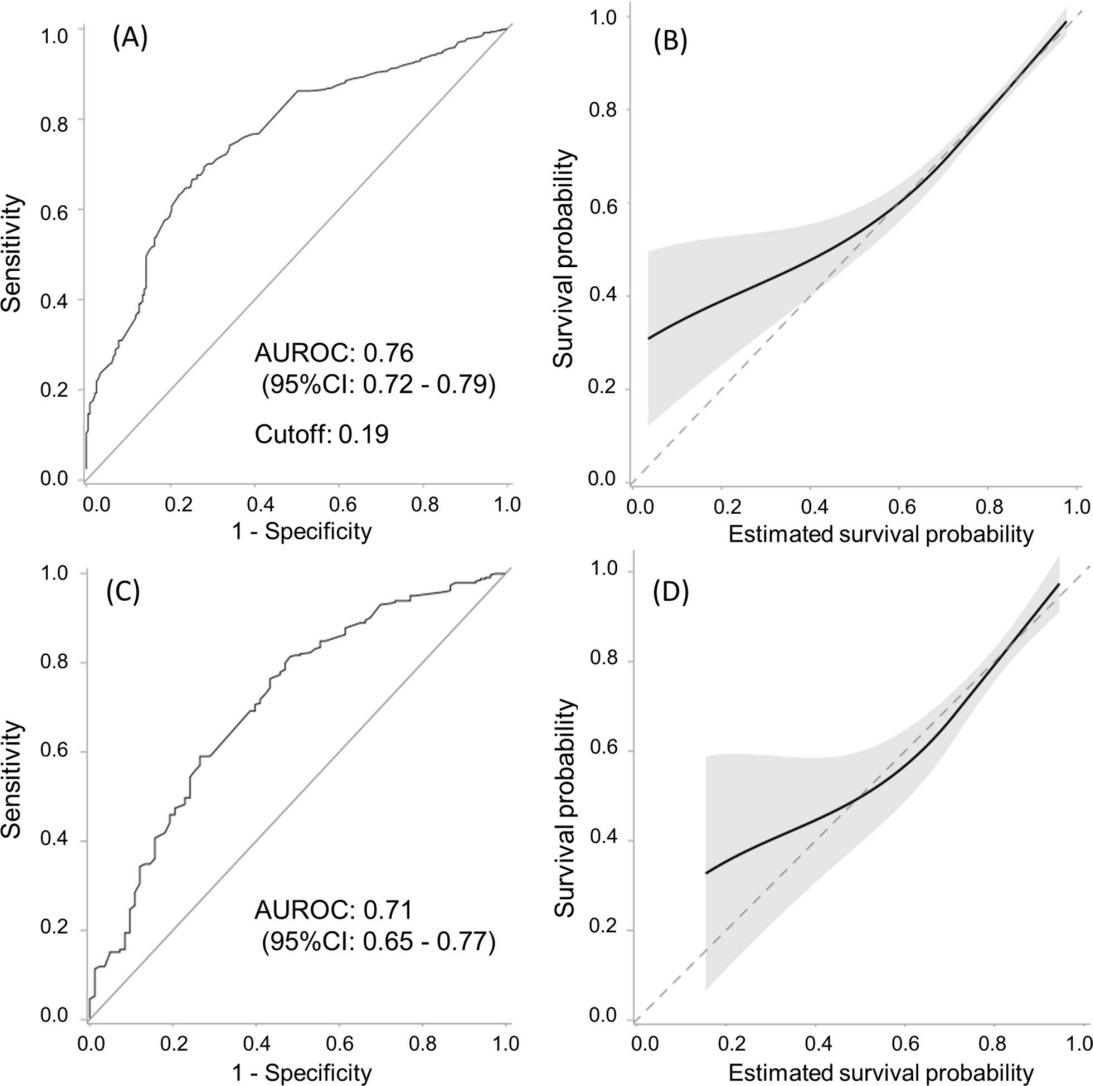
Receiver operating characteristic (ROC) curve and calibration plot. **A** ROC curve in the derivation cohort. The area under the receiver operating characteristic curve (AUROC) for the composite index to predict 28-day survival showed 0.76, and the cutoff was 0.19. The positive predictive value (PPV) of the composite index was 0.928, and the negative predictive value (NPV) was 0.316. **B** Calibration plot in the derivation cohort. The calibration plot demonstrated a perfect fit of the estimated probability of survival to survival probability when the survival probability was higher than 0.6. The gray band showed a 95% confidence interval (CI). **C** ROC curve in the validation cohort. The AUROC for the composite index to predict 28-day survival showed 0.71. The PPV of the composite index was 0.913, and NPV was 0.251. **D** Calibration plot in the validation cohort. The calibration plot demonstrated the gap between the survival probability and the estimated probability of survival, especially when the survival probability was low. The gray band showed a 95% CI

ROC analysis for the CPI’s discrimination in the derivation cohort showed an AUROC of 0.76 (95% CI: 0.72–0.79), with a cutoff at 0.19. In the validation cohort, the AUROC was 0.71 (95% CI: 0.65–0.77). Calibration plots showed approximate concordance in the derivation and validation cohorts (Fig. 2).

**Fig. 3:**
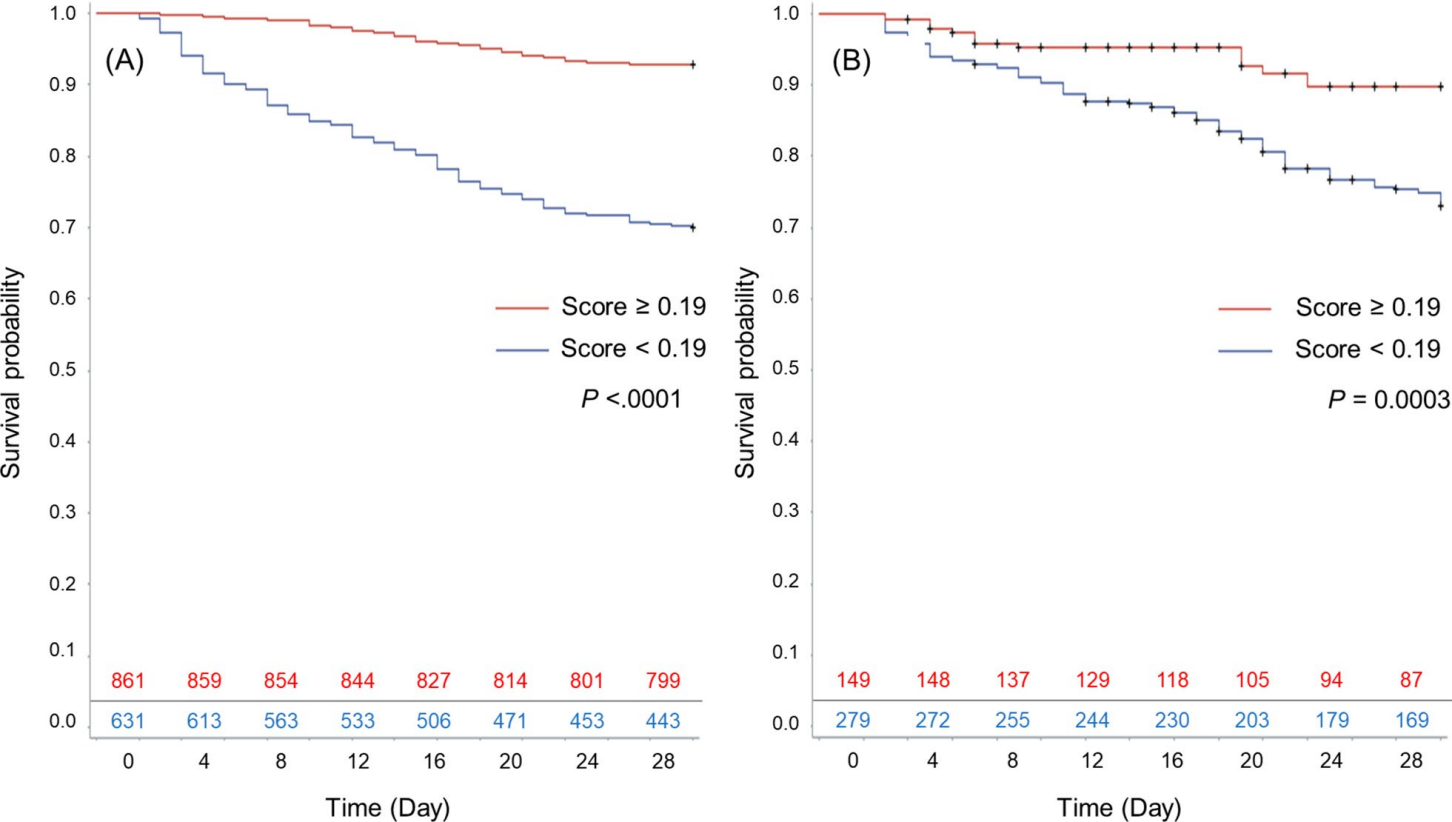
Kaplan–Meier curve stratified by index cutoff in the two cohorts. **A** Kaplan–Meier curve stratified by index cutoff in the derivation cohort. The survival rate of the septic DIC patients with a score of 0.19 and more was significantly higher than that of patients with a score of less than 0.19 (92.8% vs. 69.9%, *P* < 0.0001). **B** Kaplan–Meier curve stratified by index cutoff in the validation cohort. The survival rate of the septic DIC patients with a score of 0.19 and more was significantly higher than that of patients with a score of less than 0.19 (91.3% vs. 74.9%, *P* = 0.0003)

Kaplan–Meier survival curves showed that in the derivation cohort, patients with CPI ≥ 0.19 had a 28-day survival rate of 92.8%, significantly higher than those with a score < 0.19 (69.9%, *P* < 0.0001). The sensitivity and specificity of the CPI were 74.9% and 66.6%, respectively. Similarly, in the validation cohort, survival rates were 91.3% for scores ≥ 0.19 and 74.9% for scores < 0.19, indicating a significant difference (*P* = 0.0003) (Fig. 3). Sensitivity and specificity were 84.3% and 39.4%, respectively.

## Discussion

DIC, a complex condition often associated with sepsis, involves a vicious cycle of intravascular inflammation and microthrombosis, ultimately resulting in organ dysfunction and increased mortality [15]. Kudo et al. [16] performed a prospective multicenter observational study and reported DIC (OR: 2.71, 95% CI: 1.45–5.27) and stage 3 acute kidney injury (OR: 1.98, 95% CI 1.07–3.63) were the significant factors that associated with higher mortality. For managing sepsis-associated DIC, the efficacy of treatment with anticoagulants continues to be debated [17]; however, the use of antithrombin in this setting is primarily in Japan. Antithrombin is one of the anticoagulants recommended in the Japanese sepsis management guidelines [18]. However, the challenge in antithrombin substitution is the absence of a reliable indicator to assess its treatment effect. Therefore, the primary objective of this study was to develop a straightforward index capable of assessing the treatment effect of antithrombin at the end of a 3-day substitution.

In this study, multivariate Cox regression analysis indicated that the recovery from DIC and the rate of SOFA score change were the factors that were associated with the 28-day survival. Initially, we expected that the changes in the DIC score would be the pivotal factor related to outcomes. However, the provisional calculation did not find a valuable impact of DIC score changes. Instead, the presence or absence of DIC on Day 3 was a valuable predictor of outcomes. As for DIC recovery, Matsuoka et al. [19] reported the association between persistent DIC and poor outcomes, and they also reported better outcomes in patients who recovered in 3 days from DIC. The DIC recovery rate has also been used as the endpoint of randomized controlled studies targeting DIC [20, 21]. In the current study, the DIC recovery rate was found to be most significantly associated with 28-day survival, followed by the changes in SOFA score. Regarding the significance of adding DIC resolution, among 877 cases that were diagnosed as ‘estimated to survive’ by CPI, the rate of SOFA score change was less than 0.19 in 182 cases (20.8%). Among them, 164 cases (90.1%) actually survived, meaning that 'DIC resolution' contributed to correct changes in the results in these 164 cases.

Both post-treatment DIC and SOFA scores were significantly higher in the non-survivor group (Supple. Table S1). The SOFA score was developed to assess the severity of sepsis, and the total SOFA score is routinely employed in the ICU [22]. We compared the performance of the changes in SOFA score and the rate of SOFA score change (0.72 and 0.74, respectively) and selected the rate of SOFA score change because it demonstrated better performance. When DIC resolution is recognized, the patients are highly likely to survive. On the contrary, the rate of SOFA score change is the determinant when DIC resolution is not achieved. Therefore, the impact of the rate of SOFA score change in patients without DIC resolution on predicting 28-day survival was analyzed. As a result, it showed discriminative power as seen in CPI, and the survival curve of the rate of SOFA score change ≥ 0.19 was almost identical to that seen in patients with DIC resolution (Supple Fig. S1). The result suggested that the CPI threshold of 0.19 is substantially the rate of SOFA score change on predicting 28-day survival in patients without DIC resolution. Other than those, post-treatment antithrombin activity was also associated with survival. Akahoshi et al. [23] reported antithrombin activity ≥ 70% or 80% on day 3 was significantly associated with DIC recovery and survival rates. However, the association was less significant in our study, which is consistent with our previous study [24], and we did not include it in the CPI.

Since the objective of this study was to develop a straightforward index to assess the treatment effect after a 3-day supplementation, we evaluated the performance of DIC recovery on Day 3 and the changes in the SOFA score from Day 0 to Day 3. As a result, the significance of both factors was almost equal. Consequently, CPI became simple and calculated by DIC resolution (yes: 1, no: 0) + rate of SOFA score change. The ROC analysis revealed that the optimal cutoff value was 0.19, with an AUC of the composite CPI for predicting 28-day survival being 0.76. Kaplan–Meier curve showed the 28-day survival in cases with CPI ≥ 0.19 was 92.8% and significantly higher than that in cases with < 0.19. We think this high survival probability is crucial to use for effectiveness evaluation.

Following the delivery of the CPI, the performance of the composite CPI was validated in another cohort constructed from nationwide sepsis registry data [9]. The result showed that the AUC of CPI for predicting 28-day survival was 0.71. The performance of CPI was lower than that recognized in the derivation cohort. One reason is that the DIC recovery was judged on Day 2, and Day 2 SOFA score was used as a substitute in validation. Evaluating the predictive performance at an earlier timing might diminish CPI accuracy. Furthermore, the validation cohort had limited cases due to insufficient data availability. However, the Kaplan–Meier curve showed that the 28-day survival in cases with a CPI ≥ 0.19 was 91.3%, and the survival probability exceeded 90%, as seen in the derivation cohort. Such high probabilities were achieved because DIC was not merely a complication of sepsis, but it was deeply involved in the pathogenesis of organ dysfunction.

In summary, we found that CPI calculated by DIC recovery and the rate of SOFA score change after antithrombin treatment could predict the patient's survival on day 28. Li et al. [25] conducted a prospective study involving 209 patients with sepsis, reporting that the trajectory of sepsis-induced coagulopathy (SIC) and SOFA scores can predict prognoses. In their study, SIC and SOFA scores exhibited a gradual increase during the initial 4 days in non-survivors. In contrast, although these scores increased on Day 1, they decreased by Day 3, dropping below the levels observed on Day 0 in survivors. Therefore, the Day 3 data were considered useful to estimate the 28-day survival. Consequently, CPI can help physicians to judge the effectiveness of the treatment. When CPI is 0.19 or higher, it may not be necessary to continue the additional anticoagulation. On the contrary, if CPI was less than 0.19, changes in treatment plans should be considered. Besides its practical application, CPI may offer a more direct means of evaluating treatment effects than 28-day survival in clinical trials.

## Limitations of the study

There are several limitations in this study. First, CPI needs to be validated by newer cohorts because this study uses cohorts that were collected 10 years ago and differs from the current diagnosis criteria of sepsis and the current treatment strategy.

Second, while CPI comprising DIC resolution and rate of SOFA score change may help identify the surviving patients, it does not necessarily imply that the CPI can effectively assess the treatment effect. Since the patients with spontaneous DIC recovery (regardless of the antithrombin treatment) were included, the patients with CPI of 0.19 or more could not reflect the treatment effect. In addition, the cohorts used in the current study had limited information on concomitant medications other than antithrombin replacement. To validate the usefulness of CPI, a prospective study examining the relation between CPI score and treatment effects in septic DIC patients treated with or without antithrombin substitution is needed. Such a study would also provide valuable insights into the effectiveness of antithrombin supplementation in sepsis-associated DIC.

Third, although CPI validation should be done using a similar data set as the derivation cohort, the JSEPTIC-DIC database did not include the DIC score and SOFA score on Day 3. Therefore, the performance of CPI should be reexamined in the other study.

Finally, the performance of CPI was evaluated only in septic DIC patients treated with antithrombin, and its application was limited to a specific population. It is necessary to examine further whether this index is capable of being applied to septic DIC patients without treatment or treated with other anticoagulants.

## Conclusions

We propose a newly delivered CPI capable of estimating the 28-day survival in patients with sepsis-associated DIC. Since this index is easy to calculate and provides an earlier assessment following three days of treatment, it would be suitable for clinical practice. In addition, CPI is not affected by the factors that occur after the treatment and provides a better endpoint for clinical trials.

### Supplementary Information

**Additional file 1: Figure S1.** Kaplan–Meier curve of the patients without resolution of DIC depending on the rate of SOFA score change. Kaplan–Meier curves stratified by the rate of SOFA score changes of 0.19 or more and less than 0.19 in patients without DIC resolution on Day 3 were plotted.

**Additional file 2: Table S1.** Characteristics in the two cohorts after treatment.

## Data Availability

The validation dataset used and/or analyzed during the current study is available from the corresponding author upon reasonable request.

## Abbreviations

DIC: Disseminated intravascular coagulation
AUROC: Area under the receiver operating characteristic curve
HR: Hazard ratio
SOFA: Sequential Organ Failure Assessment
CPI: Composite prognostic index
MODS: Multiple organ dysfunction syndrome
OR: Odds ratio
JSEPTIC-DIC: Japan Septic Disseminated Intravascular Coagulation
ICU: Intensive care units
CI: Confidence interval
SIC: Sepsis-induced coagulopathy
