# Supplementary figures and images for: Determining prognostic indicator for anticoagulant therapy in sepsis-induced disseminated intravascular coagulation
Source: J Intensive Care. 2024 Jun 24;12:24. doi: 10.1186/s40560-024-00739-x (PMC11194983; doi:10.1186/s40560-024-00739-x)

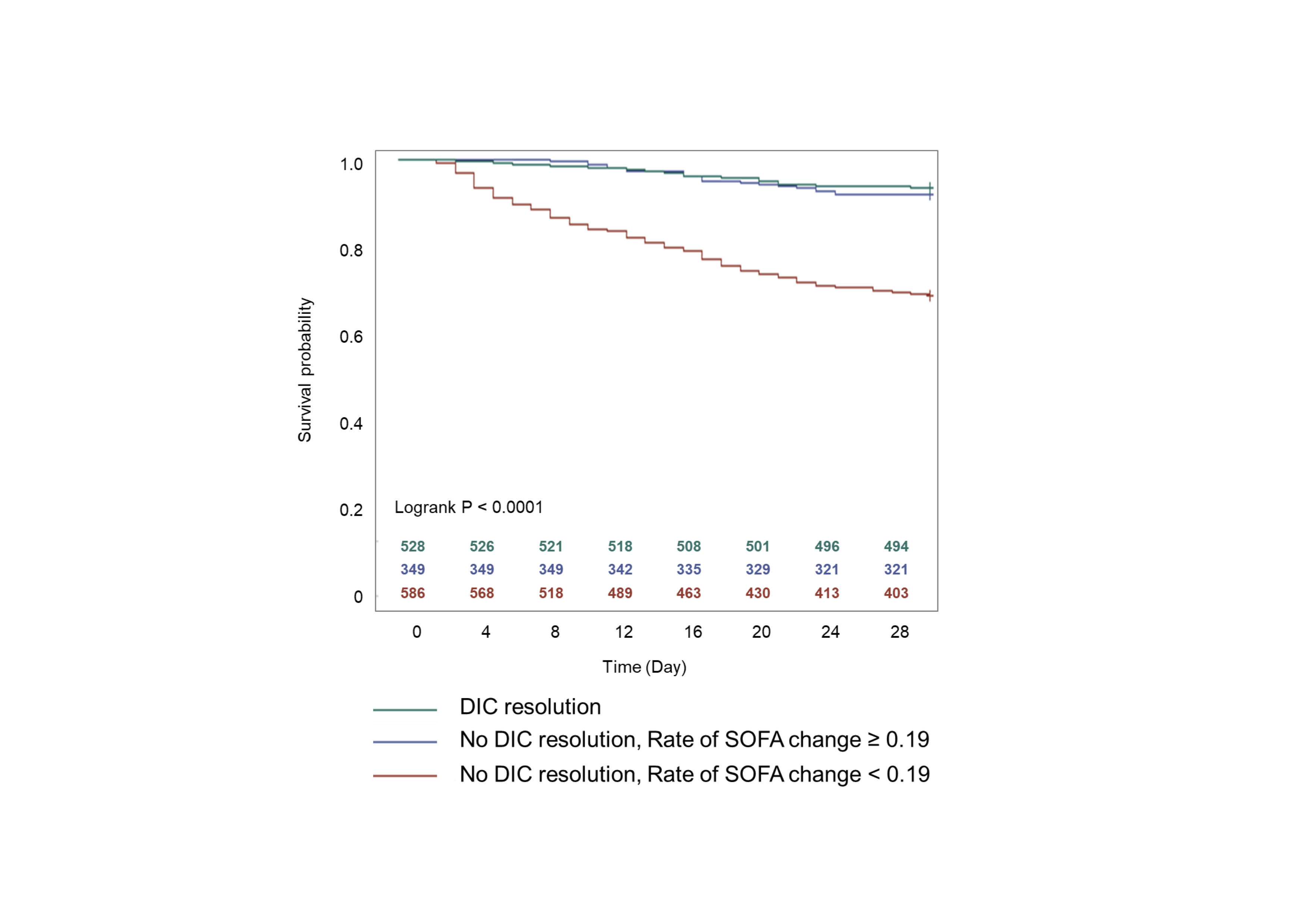

Supplement: Supplementary file 1 — Additional file 1: Figure S1. Kaplan–Meier curve of the patients without resolution of DIC depending on the rate of SOFA score change. Kaplan–Meier curves stratified by the rate of SOFA score changes of 0.19 or more and less than 0.19 in patients without DIC resolution on Day 3 were plotted. [file 40560_2024_739_MOESM1_ESM.tiff]
