# Supplementary material for: Determining prognostic indicator for anticoagulant therapy in sepsis-induced disseminated intravascular coagulation
Source: J Intensive Care. 2024 Jun 24;12:24. doi: 10.1186/s40560-024-00739-x (PMC11194983; doi:10.1186/s40560-024-00739-x)
Supplement: Supplementary file 2 — Additional file 2: Table S1. Characteristics in the two cohorts after treatment. [file 40560_2024_739_MOESM2_ESM.docx]

**Supplementary Table1. Characteristics in the two cohorts after treatment**

|  | **Derivation (Day3)** | | | **Validation (Day2)** | | |
| --- | --- | --- | --- | --- | --- | --- |
| Variable | Non-survivor n=252 (%) | Survivor n=1,240 (%) | *P* value | Non-survivor n=83 (%) | Survivor n=345 (%) | *P* value |
| JAAM DIC score | 5.2 (1.7) | 3.9 (2.0) | <.0001 | 5.6 (2.1) | 4.9 (2.0) | 0.0003 |
| SOFA score | 12.7 (4.3) | 7.1 (4.1) | <.0001 | 14.5 (4.4) | 9.9 (4.4) | <.0001 |

Continuous variables are presented as means with standard deviation (SD).

JAAM: Japanese Association for Acute Medicine, DIC: disseminated intravascular coagulation, SOFA: sequential organ failure assessment
